# *VKORC1* and *CYP2C9* Polymorphisms: A Case Report in a Dutch Family with Pulmonary Fibrosis

**DOI:** 10.3390/ijms20051160

**Published:** 2019-03-07

**Authors:** Petal Wijnen, Marjolein Drent, Otto Bekers, Johny Verschakelen, Aalt Bast

**Affiliations:** 1 Department of Clinical Chemistry, Central Diagnostic Laboratory, Maastricht University Medical Centre, 6229 HX Maastricht, The Netherlands; petal.wijnen@mumc.nl (P.W.); o.bekers@mumc.nl (O.B.); 2 ILD Care Foundation Research Team, 6711 NR Ede, The Netherlands; johny.verschakelen@uz.kuleuven.ac.be (J.V.); a.bast@maastrichtuniversity.nl (A.B.); 3 Department of Pharmacology and Toxicology, Faculty of Health, Medicine and Life Science, Maastricht University, 6200 MD Maastricht, The Netherlands; 4 ILD Center of Excellence, St. Antonius Hospital, 3435 CM Nieuwegein, The Netherlands; 5 Department of Radiology, University Hospital Gasthuisberg, B-3000 Leuven, Belgium; 6 Venlo Campus, Maastricht University, 5900 AA Venlo, The Netherlands

**Keywords:** familial idiopathic pulmonary fibrosis, vitamin K supplementation, polymorphism, oxidative stress

## Abstract

Here, we describe a Dutch family with idiopathic pulmonary fibrosis (IPF). We hypothesized that there might be an association between the presence of Vitamin K epoxide reductase complex 1 (*VKORC1*) and/or cytochrome P450 *2C9 (CYP2C9)* variant alleles and the early onset of IPF in the members of this family. *VKORC1* (rs9923231 and rs9934438) and *CYP2C9* (rs1799853 and rs1057910) were genotyped in this family, which includes a significant number of pulmonary fibrosis patients. In all family members, at least one of the variant alleles tested was present. The presence of the *VKORC1* variant alleles in all of the IPF cases and *CYP2C9* variants in all but one, which likely leads to a phenotype that is characterized by the early onset and progressive course of IPF. Our findings indicate a role of these allelic variants in (familial) IPF. Therefore, we suggest that the presence of these variants, in association with other pathogenic mutations, should be evaluated during genetic counselling. Our findings might have consequences for the lifestyle of patients with familial IPF in order to prevent the disease from becoming manifest.

## 1. Introduction

Diffuse or interstitial pulmonary diseases (ILD) can involve various stages of fibrosis [1]. After sarcoidosis, pulmonary fibrosis is one of the most common ILDs. There are many types of pulmonary fibrosis and the causes vary [2,3,4,5]. Where the origin remains unknown, as is often the case, the category has been classified as fibrosing interstitial pneumonias (IPs) or idiopathic interstitial pneumonias (IIPs) [2,3]. Idiopathic pulmonary fibrosis (IPF) accounts for the majority of lung diseases that were classified as IIP [2].

Pulmonary fibrosis is the main cause of severe morbidity and mortality in this group. For most of these diseases, a genetic basis, environmental factors, and certain triggers have been suggested as possible risk factors. In recent years, there has been major progress in the discovery of genetic factors that contribute to the disease. Various studies have found an association between genetic polymorphisms, the presence of certain variants, and the occurrence and/or progression of ILDs of unknown origin. Some forms of pulmonary fibrosis have a familial component, which can even become manifest at a relatively young age [6,7,8,9,10,11].

Although, by definition, IPF has an unknown etiology, a number of potential risk factors have been described. Pulmonary fibrosis results from a variety of insults to the lung [12]. It represents one end of a spectrum of tissue responses to injury. The resulting histopathological changes in the lung can be diverse, with overlapping features, which are characterized by varying degrees of inflammation and fibrosis [12,13,14,15]. 

Familial and sporadic IPF are clinically, histologically, and, so far, in terms of genetic alterations, indistinguishable, suggesting that the same signaling pathways may be affected in both forms of this disease. Up to 10% of IPF cases cluster in families, suggesting that, in such cases, genetic susceptibility plays a more dominant role in the pathogenesis [10,12,16,17]. So far, however, large-scale linkage attempts across multiple families have had limited success in identifying genetic links in familial IPF. This is likely due to different genes (e.g. surfactant protein C (*SFTPC*), mucin (*MUC5B*), or telomerase reverse transcriptase (*TERT*)) being involved in different families [10,11,16,17,18,19,20].

Functional gene polymorphisms have been associated with either the incidence and/or progression of IPF. Evidence for mutations that is associated with the development of pulmonary fibrosis raises numerous clinical questions. From a pathophysiological point of view, the function of the genes highlights the central roles of the alveolar epithelium and aging in fibrogenesis [10].

Hence, it is likely that the interaction between environmental factors and genetic susceptibility strongly influences the tissue injury and repair processes that culminate in fibrosis [16,21]. We previously found that the carriers of the vitamin K epoxide reductase complex 1 (*VKORC1*) and/or cytochrome P450 *2C9 (CYP2C9)* variant allele that use oral anticoagulants have a predisposition to develop diffuse alveolar hemorrhage (DAH) events [22]. It is known that acute exacerbation of IPF shows characteristics of DAH [23], and that symptoms of DAH can be reinforced by vitamin K deficiency [24].

Moreover, we recently found an association between the occurrence of DAH and the subsequent development of IP [25]. Therefore, we hypothesized that the *VKORC1* and *CYP2C9* variant alleles might be present in a family with IPF. To test this hypothesis, *VKORC1* (rs9923231 and rs9934438) and *CYP2C9* (rs1799853 and rs1057910) were genotyped in the family that we studied.

## 2. Results

This was a retrospective descriptive study. We studied a family of nine spanning three generations of Caucasian European descent, four female members of which had been diagnosed with IPF. All clinical and laboratory data were retrospectively collected from medical records. High resolution computed tomography scans were reviewed by an experienced radiologist (JV). All clinical data, including the histological pattern, were classified, and the diagnosis of IPF was confirmed in accordance with the ATS/ERS consensus classification [13]. None of the family members that were studied had had any relevant medical history and/or occupational or environmental exposition related to IPF development, before the IPF became apparent or during the subsequent course of the IPF. They did not use any medication, except oral contraceptives (family members 2 through 5). Demographic and clinical features of the members of this Dutch Caucasian middle-class family are summarized in Table 1. 

**Figure 1:**
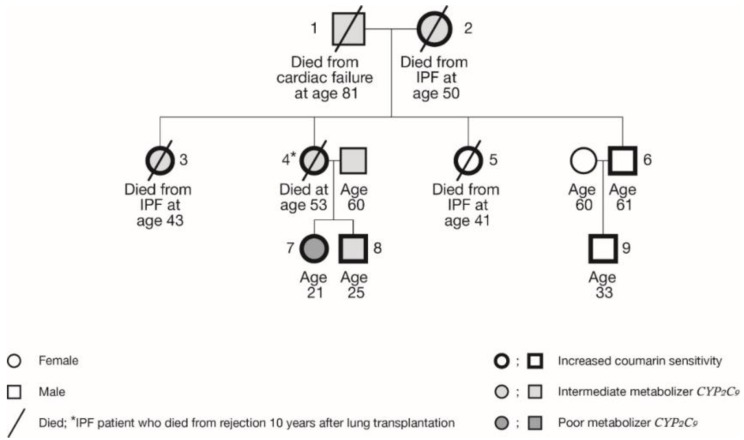
Pedigree of the studied family. Actual genotypes are listed in Table 1.

IPF had been diagnosed in four female members of this family (i.e. the mother and her three daughters) at a rather young age (Table 1, Figure 1). Family members 2, 3, and 5 died from respiratory failure due to progression of IPF, at the ages of 43, 45, and 50 years. Family member 4 successfully underwent double lung transplantation in 2003 at the age of 42. However, since a follow-up visit in June 2013, she rapidly deteriorated and then subsequently died in January 2014 at the age of 53. Family member 1 died in 2017 of heart failure, aged 81 years.

All the members of this family were found to possess at least one variant allele, so all had at least one less or severely less functional enzyme (Table 1). All four affected family members possessed at least a *VKORC1* variant allele, with three of them also carrying a *CYP2C9* allelic variant. The three grandchildren are all heterozygous for *VKORC1*, but for the *CYP2C9*, they are either *1/*1 (no variant allele, family member 9), *1/*2 (heterozygous, family member 8), or *2/*2 (homozygous variant alleles, family member 7). Until now, the third generation has not shown any signs of pulmonary fibrosis.

## 3. Discussion

The presence of both a *VKORC1* and a *CYP2C9* variant allele in all but one of the IPF patients in the family that we studied is striking. A frequent single nucleotide polymorphism (SNP) within the *VKORC1* promoter (G-1639A) has been identified as a major determinant of coumarin sensitivity, reducing vitamin K epoxide reductase enzyme activity to 50% of the wild GG type. Variant allele carriers are at an increased risk of bleeding events when using oral anticoagulants or antibiotics, and/or through contact with triggers that have similar vitamin K antagonistic properties [22,28]. Therefore, it is tempting to speculate that the interplay between certain environmental factors and/or food and drug use, together with genetic susceptibility, influences the tissue injury and repair processes that culminate in fibrosis [12].

According to current views, the occurrence of DAH episodes as well as functional gene polymorphisms of a number of cytokines might also play a role in the pathogenesis and deterioration of fibrosing IPs. Oxidative damage has been suggested to be a trigger in the pathophysiology of IPs. Bleeding or subclinical bleeding events release iron into the lung, and this free toxic iron causes oxidative stress, inflammation, and finally irreversible damage or fibrosis [29,30]. This is further borne out by the observation that stimulators of reactive oxygen species (ROS), such as angiotensin II or bleomycin, are initiators of pulmonary fibrosis [30]. Since DAH leads to huge oxidative stress, it might trigger, cause, or strengthen the development of pulmonary fibrosis. This hypothesis has been tested in a group of 65 patients who had had at least one confirmed episode of DAH [25]. Of these 65 patients, 31 (48%) eventually turned out to have developed a fibrosing IP within three years after confirmation of the diagnosis of DAH. Twenty-two of those who died (54%) eventually turned out to have developed a fibrosing IP. This study supported the hypothesis that DAH is a potential cause or trigger of this disorder [25]. In a cohort of patients with pulmonary fibrosis, it was found that a considerable percentage had developed fibrosis after starting anticoagulant therapy. Moreover, this group possessed a higher frequency of *VKORC1* and *CYP2C9* variant alleles, and many patients had an unstable International Normalized Ratio (INR) and more often had to adjust their dosage of anticoagulants [25]. To date, *VKORC1* and *CYP2C9* variant alleles have been associated with DAH in patients while using oral anticoagulation therapy [22]. 

The association with *VKORC1* and/or *CYP2C9* variant alleles might even be a risk factor for the development or exacerbation of pulmonary fibrosis. While the *VKORC1* and *CYP2C9* SNPs, as such, are rather common with genotype frequencies of around 45% for *VKORC1* GA, 15% for *CYP2C9* *1/*2, and 10% for the *CYP2C9* *1/*3 variant, their simultaneous occurrences can range from around 5 to 10% in Caucasians [31]. A simultaneous occurrence of *VKORC1* and *CYP2C9* variant alleles appears to increase the vulnerability to glitches in the vitamin K cycle, as well as increasing the probability of developing or stimulating progression of IPF.

Patients with IPF also frequently suffer from infections, and these episodes are associated with disease exacerbation and progression [23]. It is well known that antibiotic treatment and/or malnutrition are risk factors that can induce hemorrhages, which is probably due to direct interference with the vitamin K metabolism, causing a relative deficiency [28,32,33]. Hence, antibiotics that were used during IPF exacerbations might be a cofactor in progression, especially in patients carrying variant alleles. Besides the metabolic influences on vitamin K, the antibiotic nitrofurantoin, for instance, is an ROS generator, as it produces superoxide radicals via redox cycling [34]. It has been found that superoxide radicals enter the lung fibroblasts through chloride channels, which can then lead to the generation of transforming growth factor beta (TGF-β) and collagen formation [35]. Thus, redox cycling may be critically involved in the etiology of pulmonary fibrosis. 

The risk of a clinically relevant vitamin K deficiency is increased by various circumstances, such as the use of oral anticoagulants or antibiotics, as well as reduced vitamin K uptake by the intestines, due to a variety of causes, such as fat uptake inhibitors. Differences in vitamin K metabolism due to the use of vitamin K antagonists or genetic variation can cause reduced vitamin K status [28]. Hence, simple vitamin K supplementation may prevent a relative or actual vitamin K deficiency that is caused by malnutrition/low dietary intake and/or antibiotics. Additionally, since vitamin K can act as an anti-oxidant, it reduces oxidative stress that is caused by lipopolysaccharides or by iron released during a DAH episode [36,37].

**Figure 2:**
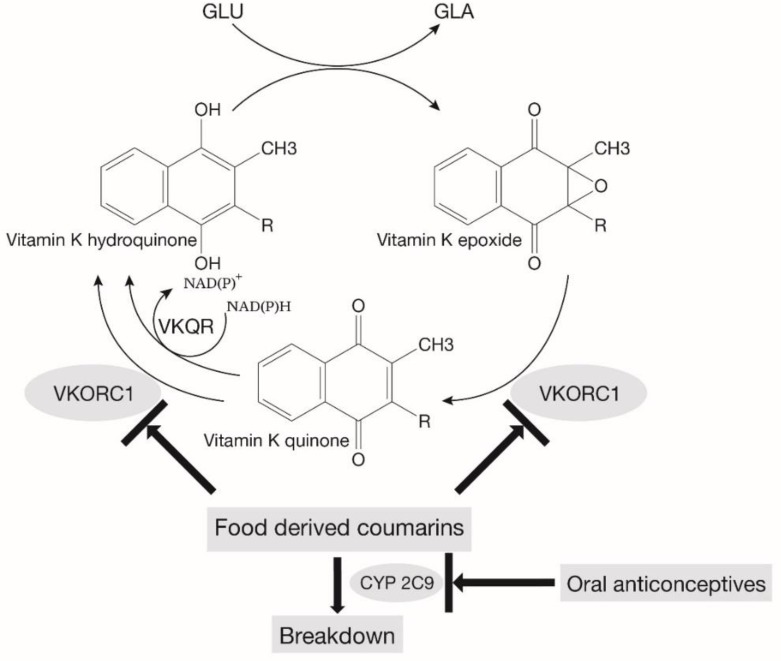
Vitamin K cycle with influences. The role of vitamin K epoxide reductase (VKORC1) and the cytochrome P450 iso-enzyme 2C9 (CYP2C9) in coagulation: VKORC1, as well as the vitamin K quinone reductase (VKQR), is involved in reducing steps in the generation of the active hydroquinone form of vitamin K. Inhibition or diminished activity of CYP2C9 leads to a decreased breakdown and subsequent excretion of food-derived VKORC1-inhibiting coumarins. Presence of polymorphisms and inhibition of VKORC1 reduces vitamin K activity, viz. carboxylation of glutamic acid (GLU) into calcium-binding gamma-glutamic acid residues (GLA). In addition, polymorphisms and inhibition of CYP2C9 by oral contraceptives or antifungal agents might decrease the breakdown of VKORC1 inhibiting compounds. Decreased VKORC1 activity has been associated with the occurrence of fibrosis.

Humans are continuously exposed to coumarins through their diet [38]. These natural coumarins inhibit the vitamin K cycle (Figure 1). Since specific variant alleles in the *VKORC1* and *CYP2C9* genes, and in the case of acenocoumarin also in the *CYP2C19* gene, are associated with low vitamin K recycling rates and a low vitamin K status, knowing that the genotype can help in predicting a patient’s response to warfarin or clopidogrel [39,40]. In the presence of such variant alleles, the CYP2C9 and VKORC1 enzymes are less active and therefore even more sensitive to coumarins [28]. Recently, we found a history of unstable INR in an IPF patient, who possessed variant alleles for both (*CYP2C9* heterozygote and *VKORC1* homozygote) [28]. The INR stabilized and the deterioration of the lung function stopped after vitamin K supplementation. Another cause of bleeding or subclinical bleeding events or the production of ROS and possible subsequent pulmonary fibrosis are drugs that inhibit the CYP2C9 enzyme. This is the case with oral contraceptives, which could thus indirectly affect the coagulation, even when no variant alleles are present in this CYP gene (Figure 2) [41].

Next to oral contraceptives, naturally produced estrogens may also have an influence, as their metabolites are found to participate in pro- and anti-inflammatory processes, e.g. by stimulating ROS production and DNA damage [42]. Moreover, estrogen may also affect the oxidative status of cells by influencing the scavenging of free radicals [42]. Therefore, a potential treatment strategy could involve vitamin K supplementation and possibly refraining from oral contraceptive use.

At present, the third generation female member of the family that we studied is not using contraceptives and it shows no signs of pulmonary fibrosis. However, it is tempting to speculate that, since all of the affected persons were young females, nevertheless there might be a link between variant alleles and the development of pulmonary fibrosis, with a possible aggravating role for estrogens. Family member 7 is likely to be even more vulnerable to the effect of coumarins and other substances influencing the vitamin K cycle, since, in addition to an increased coumarin sensitivity, since she is a poor metabolizer for CYP2C9 and has hardly any enzyme activity to remove these compounds. Therefore, it will be necessary to monitor her very closely in order to establish any respiratory functional impairment at an early stage.

So far, there are limited therapeutic options available to treat fibrosing processes in the lung. While the available antifibrotic agents for IPF slow down its progression, they cannot repair the damage [43]. For many other pulmonary fibrosing processes, no effective therapy is available. Hence, treatment focuses on ‘supportive care’, for instance, the use of effective antacids, prevention of infections—including vaccination against pneumococci—and the use of antioxidants. Noth and colleagues have established that warfarin does not benefit patients with IPF and that their use can even be deleterious and increase mortality rates. Therefore, they recommended not giving prophylactic anticoagulants to patients with IPF [44]. In line with this, Kreuter et al. more recently demonstrated the unfavourable effects of oral anticoagulants on survival in IPF [45]. 

The importance of vitamin K for the prevention of tissue degeneration due to hardening and degradation of collagen and elastin, and the prevention of ectopic calcification, is becoming increasingly clear [46]. Unfortunately, the potential favourable contribution of vitamin K to slowing down the progression of pulmonary fibrosis has so far been insufficiently examined. It may be beneficial to determine the vitamin K status and the relevant polymorphisms of patients with fibrosing IPs, including IPF (where increased vulnerability of the alveolar epithelium plays a crucial role in the pathophysiology), and to offer dietary recommendations that aimed at improving their vitamin K status. Vitamin K supplementation is intended to counteract fluctuations that result from reduced uptake or intake. In addition, dietary advice can help to supplement vitamin K in a natural way. The vitamin K in food minimizes oxidative stress, a risk factor in the development of fibrosing IPs [28]. In addition to the causes that are described above, the inhibition of CYP2C9 and CYP2C19 can also play a role, as CYP2C9 plays a key part in the vitamin K cycle as well as in the detoxification of many xenobiotics and drugs in the broadest sense, including cocaine, marihuana, and their metabolites [47]. It has been known for quite some time that food-derived coumarins may constitute a risk to human safety [48]. New analytical chemistry methods are being continuously developed to optimize the quantification of coumarins in food [49]. The inhibition of CYP2C9 by coumarins from plants or from alternative medicines has been widely studied [50,51]. 

## 4. Materials and Methods

The DNA samples were available from all family members across three generations, except for the already deceased mother (family member 2). DNA was obtained from venous EDTA anti-coagulated blood, isolating according to the manufacturers’ instructions. The CYP2C9*2 (C430T), CYP2C9*3 (A1075C), *VKORC1* (G-1639A), and *VKORC1* (C1173T) SNPs were genotyped while using real-time PCR Fluorescence Resonance Energy Transfer analyses, as previously described [22,52].

Written informed consent was obtained from each participating individual, and the Ethics Committee of the University of Maastricht approved the study and it was performed in accordance with the principles that are embodied in the Declaration of Helsinki.

## 5. Conclusions

The presence of *VKORC1* and *CYP2C9* allelic variants in the family members suffering from IPF indicate a previously unsuspected link between these variants and pulmonary fibrosis. The existence of this link triggers new thoughts regarding the possible pathophysiologic mechanisms underlying (familiar) pulmonary fibrosis. 

The fact that disease susceptibility in this family might be—at least partly—linked to these allelic variants represents important information for (adjuvant) treatment and future clinical surveillance. As there is, at this moment in time, no effective therapy for people with a severe form of pulmonary fibrosis, we should put much more effort into preventing the development of this progressive disorder. Efforts should concentrate on protecting patients against exacerbations, and thus improving their quality of life. One potential trigger of fibrosing interstitial pneumonias is DAH, which has been associated with the presence of variant alleles in *VKORC1*, *CYP2C9*, and *CYP2C19* genes. In addition, DAH has been associated with vitamin K deficiency. Whether vitamin K supplementation could prevent exacerbations, such as diffuse alveolar damage (DAD) or DAH, which usually cause an acute clinical crisis, or could slow down the disease progression as much as possible needs to be explored in future studies.

## Figures and Tables

**Table 1:** Characteristics of the studied family.

Family Member	Year of Birth	Age (yr) at Diagnosis of IPF	Age (yr) Deceased	Current Age (yr)	Sex	*CYP2C9*	MetabolicfunctionCyp2c9 Enzyme	*VKORC1*		Coumarin Sensitivity VKORC1 Enzyme
1	1934	NA	81	NA	m	*1/*2	IM	CC	GG	normal
2	1933	39	50	NA	f	*1/*3	IM	*TT*	*AA*	high
3	1959	38	43	NA	f	*1/*3	IM	CT	GA	increased
4	1960	25	53	NA	f	*1/*2	IM	CT	GA	increased
5	1964	41	45	NA	f	*1/*1	EM	CT	GA	increased
6	1957	NA	NA	61	m	*1/*1	EM	CT	GA	increased
7	1997	NA	NA	21	f	*2/*2	PM	CT	GA	increased
8	1993	NA	NA	25	m	*1/*2	IM	CT	GA	increased
9	1985	NA	NA	33	m	*1/*1	EM	CT	GA	increased

Abbreviations: yr = years; m = male; f = female; IPF = idiopathic pulmonary fibrosis; NA = not applicable; IM = intermediate metabolizer; EM = extensive metabolizer; PM = poor metabolizer; MAF = minor allele frequency; SNP = single nucleotide polymorphism. SNPs investigated: *CYP2C9* = cytochrome P450 2C9*2 (rs1799835, C430T, MAF11.7%) and 2C9*3 (rs1057910, A1075C, MAF5.6%) [26]; *VKORC1* = vitamin K epoxide reductase complex 1 (rs9934438, C1173T, MAF14.2%) and (rs9923231, G-1639A, MAF14.2%) [27]; *1/*1, CC and GG = genotype notation of a fully functional enzyme of *CYP2C9* and *VKORC1*, respectively. Note: the genotype results for family member 2 were obtained by deduction.
